# Two bits dual-band switchable terahertz absorber enabled by composite graphene and vanadium dioxide metamaterials

**DOI:** 10.1038/s41598-024-56349-y

**Published:** 2024-03-09

**Authors:** Saeedeh Barzegar-Parizi, Amir Ebrahimi, Kamran Ghorbani

**Affiliations:** 1 Electrical Engineering Department, Sirjan University of Technology, Sirjan, Iran; 2 School of Engineering, RMIT University, Melbourne, Australia

**Keywords:** Materials science, Nanoscience and technology, Optics and photonics, Physics

## Abstract

This article presents the design of a 2-bit dual-band switchable terahertz absorber using a stacked combination of graphene and vanadium dioxide (VO_2_) metamaterials. For the first time, the proposed absorber design offers four switchable states by controlling the conductivity of graphene and VO_2_ metamaterial layers. The lower absorption band is produced by the graphene metamaterial, whereas the upper band is implemented by the VO_2_ metamaterial pattern. The structure shows two absorption bands (State 11) at 0.745–0.775 THz and 2.3–5.63 THz, when the Fermi graphene level of graphene is 0.2 eV and the VO_2_ is in the metallic phase. The lower absorption band is turned off, while keeping the upper band (State 01), when the graphene Fermi level is 0 eV and the VO_2_ layer is in the metallic phase. The upper absorption band is turned off, while preserving the lower absorption band (State 10) by switching the VO_2_ into the insulator phase and keeping the graphene Fermi level at 0.2 eV. Finally, both of the absorption bands are turned off by setting the graphene Fermi level to 0 eV and switching the VO_2_ into the insulating phase. Equivalent circuit modelling analysis and full-wave electromagnetic simulations are used to explain the operation principle of the proposed absorber. Very good agreement is obtained between the theoretical analysis and the simulations confirming the presented design principle for the 2-bit switchable absorber.

## Introduction

Absorbers are fundamental components in various telecommunication, sensing, and imaging systems operating in a broad range of spectrum from microwave up to terahertz and optical frequencies^1–15^. Metamaterials-inspired structures are attractive choices in many of such applications due to their thin profiles, near perfect absorption, and flexibility in designing both narrow and wideband absorption characteristics^1–7^. Metamaterial absorbers are conventionally implemented in three-layer configurations made of metamaterial patterned layer, a dielectric spacer and a bottom metallic layer that acts as a perfect reflector. Such a configuration has been applied in the design of absorbers with narrowband^5–7^, broadband^1–4^, and multi-band^6,7^ absorption characteristics. However, designing absorbers with tunable/reconfigurable responses still remain challenging at terahertz frequencies mainly due to the limited devices and materials that can be applied with switchable and/or tunable electromagnetic properties terahertz frequencies. Recently, tunable metamaterials using graphene^16–19^, vanadium dioxide (VO_2_)^20–22^, doped silicon^23^, germanium antimony telluride (GST)^24–26^, and liquid crystals^27,28^ were introduced to realize reconfigurable absorbers. However, in such structures just a single characteristic of the response such as center frequency, bandwidth, or absorption level can be tuned without any multi-functional or reconfigurable capability. Graphene is a unique thin two-dimensional (2D) material composed of carbon atoms, which exhibits exceptional electrical and optical properties such as tight field confinement, high electron mobility, and flexible tunability^29,30^. The conductivity of graphene can be dynamically tuned by adjusting the Fermi energy level through chemical doping or electrostatic gating^31–34^. Hence, graphene-based metamaterials are ideal candidates for tunable and switchable electromagnetic devices. On the other hand, VO_2_ is a phase change material that is highly attractive for realizing switchable and tunable devices. VO_2_ undergoes a reversible transition behavior from the insulator to the metallic phase on a sub-picosecond timescale triggered by electrical, thermal, or optical excitations. VO_2_ shows an insulating behavior in the monoclinic phase below the transition temperature (around 340 K) and a metallic behavior at the tetragonal rutile phase above it^35–45^. Thus, combination of the VO_2_ and graphene properties in a single design would potentially be an appropriate approach for realizing multi-functional switchable and reconfigurable absorbers.

Recently, tunable and switchable terahertz absorbers were developed based on hybrid metamaterials of graphene and phase change materials^46–49^. In Ref.^46^, a switchable metamaterial absorber was designed using VO_2_ and graphene. The functionality of this absorber can be switched from a broadband to a narrowband absorber by the phase transition of VO_2_ from insulator to metal. A dynamically switchable dual-broadband absorber based on a hybrid metamaterial with vanadium dioxide and graphene was designed at terahertz regime in Ref.^47^. When the vanadium dioxide is in the metallic phase and the Fermi energy level of graphene is set to zero, a high-frequency broadband absorption from 2.05 THz to 4.30 THz was achieved. This structure acts as a low-frequency broadband absorber from 1.10 THz to 2.30 THz if the vanadium dioxide is in the insulating phase. In addition, minimum absorption is observed by tuning the Fermi energy level. In Ref.^48^, a switchable graphene-vanadium oxide metamaterial-based narrowband absorber is designed. When the VO_2_ is in an insulating phase, the absorption spectrum shows a single narrowband absorption peak with near 100% absorption. When the VO_2_ is in a metallic phase, the absorber shows two narrowband absorption peaks with around 90% absorptivity. Furthermore, a wide absorption band can also be achieved by tuning the Fermi energy level.

In all of the previous works, the operational states are limited to three, whereas by having two switchable materials (i.e. graphene and VO_2_), potentially four operational states are achievable by switching the VO_2_ and graphene patterns independently. Here, using the electrical tunable properties of graphene and the phase change behavior of VO_2_, a 2-bit (four state) switchable THz dual-band (narrowband-wideband) absorber with polarization insensitive^46–52^ and high angular response stability is designed. The structure includes two layers of graphene patterned square and VO_2_-patterned elements of cross strips. The resonant behavior of the graphene layers is used to realize a low-frequency narrow absorption band, while the resonant properties of VO_2_ layer in the metallic phase is used to obtain a broadband absorption at the higher frequency range. Thus, the structure shows dual narrow-wide absorption bands, when the Fermi level energy of graphene is set to 0.2 eV and VO_2_ is in the metallic phase (state 11). When the Fermi energy level of graphene is 0.2 eV and VO_2_ is switched to the insulator phase, the absorber shows a single narrow absorption band at low frequencies (state 10). By changing the Fermi energy level to 0 eV, the lower frequency absorption band is omitted, and the absorber shows a single high frequency absorption band in metallic phase of VO_2_ (state 01). In the fourth state, when the Fermi energy level of graphene is 0 eV and VO_2_ is switched to the insulator phase, there is no absorption band (state 00). The design concept can also be extended to a dual wideband switchable absorber by tuning the relaxation time of graphene and change in thickness of dielectric spacer embedded at the bottom of the graphene layer. A similar switching scenario can be employed for the wideband/wideband prototype as well.

The rest of the paper is organized as follows: Section "Structural design" describes the configuration of the proposed absorber and an equivalent circuit model. The absorption performance of the designed absorber is investigated in Section "Results and discussion (dual narrowband-broadband absorber)" together with its four-state switchable behavior. In Section "Dual broadband switchable absorber", a dual wideband/wideband absorber is proposed as an extension of the presented design. Finally, Section "Conclusion" presents the main conclusions.

## Structural design

**Figure 1:**
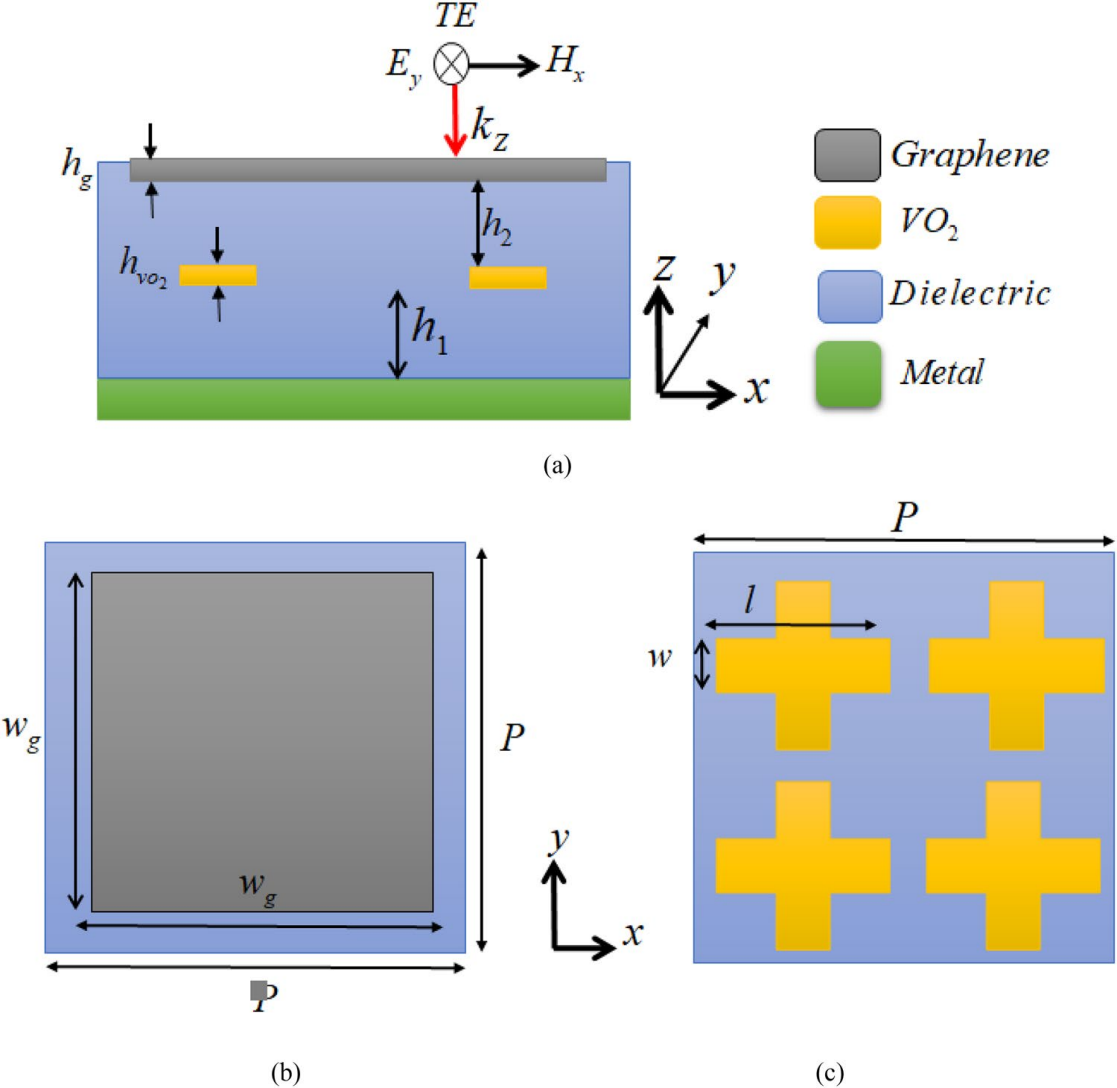
(**a**) Cross-sectional view of a unit cell including: a top layer of a graphene patch, first layer of the dielectric spacer, a layer of four VO_2_ cross strips, second layer of the dielectric spacer, and a bottom metallic layer. Directions of the E and H-fields are presented for the TE mode of the incident wave. Top views of (**b**) the graphene patch and (**c**) the VO_2_ cross strips.

A uint cell of the proposed absorber including the two layers of resonators patterns seperated by a dielectric spacer is shown in Fig. 1. From top to the bottom, the absorber composes of a graphene layer of thickness *h*_*g*_, a dielectric spacer of thickness *h*_2_, four VO_2_ cross strips with a thickness of *h*_VO2_, a dielectric spacer with a thickness of *h*_1_, and the bottom metallic film of thickness *t*_*m*_. The graphene layer is designed as an array of square patchs of width *w*_*g*_, and the VO_2_ layer includes an array of VO_2_-patterned elements of cross strips, where the length and width of the VO_2_ strips are *l* and *w*, respectively. The dielectric spacer is Polyethylene Cyclic Olefin Copolymer (COC) with a relative permittivity of *ε*_r_ = 2.3 and tan*δ* = 0.0006. The periodicity in both *x* and *y* directions is *P.* The bottom metal layer is gold. The thickness of the bottom metallic layer is larger than the skin depth. Thus, it acts as a perfect reflector and a perfect electric conductor (PEC) approximation is valid. Figure 1a also shows directions of the E and H-fields in incident electromagnetic wave for the TE mode. The E and H-fields in the TE mode are in *y* and *x*-directions, respectively. Because of the structural symmetry, the design works for the TM mode as well, where in the TM mode the E and H-fields directions are in the x and y-directions, respectively. In the absorber design, the VO_2_ is placed at the bottom since the lower absorption band is produced by the graphene metamaterial layer. The graphene layer requires a thicker dielectric spacer since it produces absorption at the lower frequency band. At these frequencies, the VO_2_ layer is almost transparent to the incident electromagnetic fields and thus the combination of graphene layer and the thick dielectric spacer formed by a combination of *h*_1_ and *h*_2_ dielectric spacers result in the lower absorption band.

**Figure 2:**
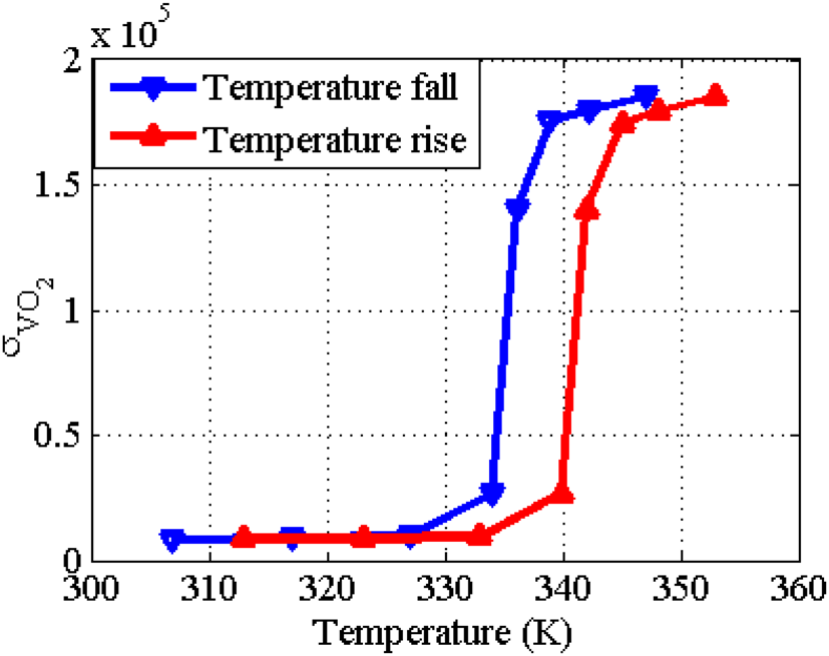
The conductivity of VO_2_ as function of temperature extracted from data presented in Ref.^51^.

**Figure 3:**
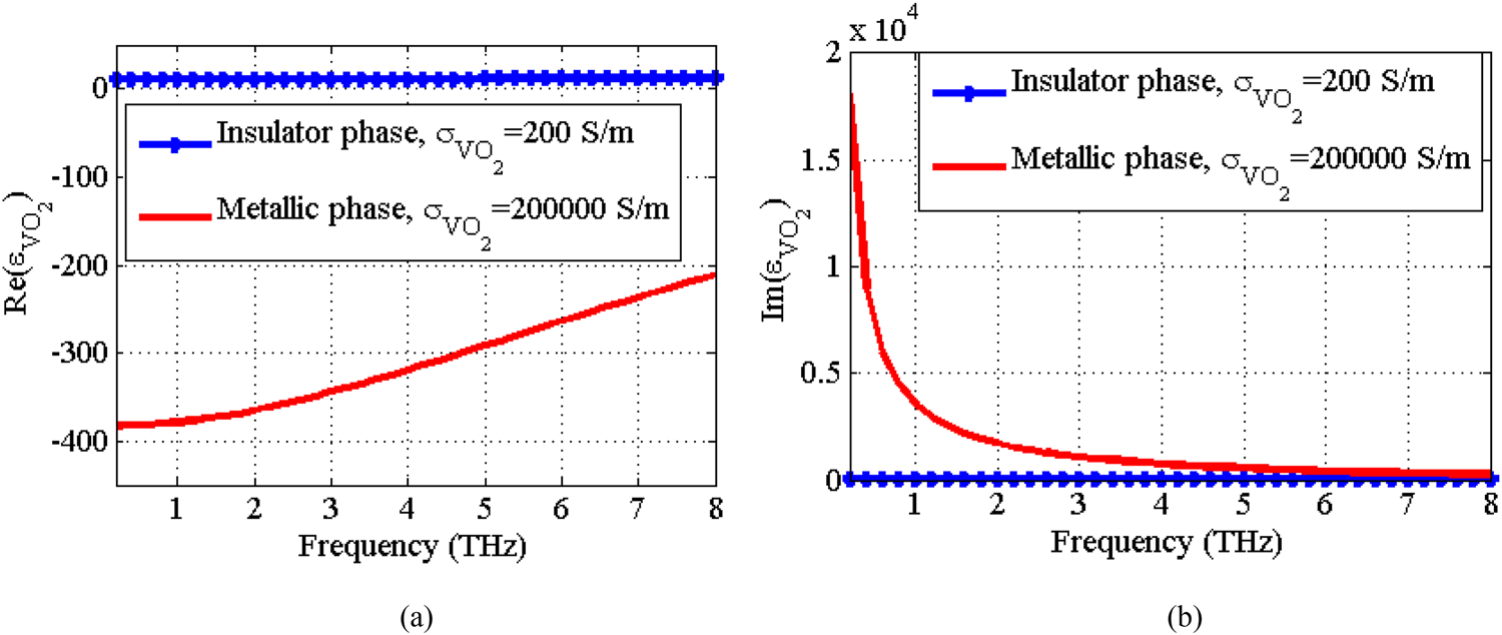
The complex permittivity of VO_2_ as function of frequency for two stable phases as dielectric phase (*σ*_VO2_ = 200 S/m) and metallic phase (*σ*_VO2_ = 200,000 S/m). (**a**) Real part of the permittivity and (**b**) the imaginary part of the permittivity.

Within the THz range, the relative permittivity of VO_2_ can be expressed using the Drude model^35^:

1 $$ \varepsilon_{{VO_{2} }} (\omega ) = \varepsilon_{\infty } - \frac{{\omega_{p}^{2} (\sigma_{{VO_{2} }} )}}{{(\omega^{2} + i\gamma \omega )}} $$

where $\varepsilon_{\infty } = 12$ is the permittivity at very high frequencies,$\omega_{p}^{2} (\sigma_{{VO_{2} }} ) = \left( {{{\sigma_{{VO_{2} }} } \mathord{\left/ {\vphantom {{\sigma_{{VO_{2} }} } {\sigma_{0} }}} \right. \kern-0pt} {\sigma_{0} }}} \right)\omega_{p}^{2} (\sigma_{0} )$ is the plasma frequency with $\omega_{p} (\sigma_{0} ) = 1.4 \times 10^{15} Rad/s$, $\gamma = 5.75 \times 10^{13} \,\,Rad/s$ is the damping rate, and $\sigma_{0} = 3 \times 10^{5} \,S/m$. VO_2_ is a temperature sensitive material that transits from an insulator to metal at a critical temperature around 340 K. The variations of the VO_2_ conductivity as a function of the temperature at both of the temperature rising and falling cycles are extracted from Ref.^51^ and plotted in Fig. 2. As observed, the temperature change drastically affects the conductivity of VO_2_, especially at the phase transition point. Due to the thermal hysteresis effect, the transition curves are slightly different in the rising and falling cycles, however, it does not affect the transition between two stable phases. By using the plot in Fig. 2 and Eq. (1), one can plot the relative permittivity of VO_2_ at different temperatures. Figure 3 shows the relative permittivity as function of frequency for the insulator phase ($\sigma_{{VO_{2} }} = 200\,S/m$) and the metallic phase ($\sigma_{{VO_{2} }} = 200000\,S/m$). The conductivity of VO_2_ ($\sigma_{{VO_{2} }}$) can be tuned by applying thermal or electrical stimuli^39–43^.

The surface conductivity of graphene is described by the Kubo equation^52^:

2 $$  \begin{aligned}   \sigma _{g} (\omega )\, &  = \,\sigma _{{\text{int} er}}  + \sigma _{{\text{int} ra}} \, =  \\     & \frac{{2e^{2} k_{B} T}}{{\pi \,\hbar ^{2} }}\frac{j}{{ - \omega  + j\tau ^{{ - 1}} }}\ln \left[ {2\cosh \left( {E_{f} /2k_{B} T} \right)} \right] \\     &  - \frac{{je^{2} }}{{4\pi \,\hbar }}\ln \left[ {\frac{{2E_{f}  - \hbar (\omega  - j\tau ^{{ - 1}} )}}{{2E_{f}  + \hbar (\omega  - j\tau ^{{ - 1}} )}}} \right]\, \\  \end{aligned}   $$

where *e* is the electron charge, *E*_*f*_ denotes the Fermi level, *ħ* refers to the reduced Plank constant, $k_{B}$ represents the Boltzmann constant, $\omega = 2\pi \,f$ is the angular frequency, *T* = 300 K is the temperature, and $\tau$ is the relaxation time. The surface conductivity of graphene contains intra-band conductivity (first term in (1)) and inter-band conductivity (the second term in (1)). At low frequencies, the inter-band term of conductivity is dominant and for $E_{f} > > K_{B} T$, $\sigma_{g}$ is driven as the Drude form:

3 $$ \sigma_{g} (\omega ) = \frac{{e^{2} E_{f} \tau }}{{\pi \,\hbar^{2} }}\frac{1}{1 + j\omega \tau }. $$

In simulations, graphene is modeled by a layer of thickness* h*_*g*_ = 1 nm, whose permittivity is $\varepsilon_{g} = \varepsilon_{0} - j\left( {\sigma_{g} (\omega )/h_{g} \omega } \right)$. The conductivity of graphene can be dynamically tuned by adjusting the Fermi energy level. We consider two Fermi energy levels for switching the graphene layer. For each Fermi energy level, the corresponding permittivity is extracted and then, the values are imported into the High-Frequency Structure Simulator (HFSS) software. In addition, the VO_2_ layer is modelled as a layer of thickness* h*_*vo2*_ whose relative permittivity is defined in (1). The permittivity of the VO_2_ can be controlled through the thermal excitation. When temperature is 300 K, the VO_2_ layer is in the dielectric phase and the conductivity is around 200 S/m and when temperature is 350 K, the VO_2_ layer is in the metallic phase with a conductivity around 200,000 S/m. For these two phases with different conductivities, the relative permittivity is obtained from (1) and the values are imported into the simulation software. For simulation, a unit cell of the absorber is considered with the periodic boundary conditions (PBC) along *x*- and *y*-directions and the Floquet port in *z*-direction.

The absorption is expressed as $A(\omega ) = 1 - R(\omega ) - T(\omega )$ where $R\left(\upomega \right)={\left|{S}_{11}\right|}^{2}$ and $T\left(\omega \right)={\left|{S}_{21}\right|}^{2}$ describe the reflectance and transmittance respectively, and *S* denotes the scattering parameter. The bottom metallic film is considered sufficiently thick (larger than the skin depth) with near zero transmittance. Thus, in order to achieve maximum absorption, the reflectance must be minimum. To reach this goal, it is necessary to match the input impedance of the proposed structure with the free space characteristic impedance.

The designed metasurface can be fabricated using the micro/nano fabrication technology. The fabrication process can be described as the following. In the first step, the ground plane is realized by gold coating (with 200 nm thickness) of a silicon wafer using an electron beam evaporator. Then the COC dielectric spacer will be spin coated on the gold layer. In the third step, we deposit the VOx film on the COC polymer layer through DC magnetron sputtering technique^53,54^. Then annealing in low O2 pressure will be used to convert the VOx into VO_2_. Next, the VO_2_ resonator array will be realized by a photolithography process followed by chemical etching. Again the layers of COC and polysilicon should be spin coated on VO_2_ layer. The polysilicon layers can be deposited using Low Pressure Chemical Vapor Deposition (LPCVD) process. The graphene layer can be prepared using chemical vapour deposition techniques and it will be transferred to the surface of polysilicon layer by the wet transfer technology^55–57^. In experiments, a hot plate heater placed on the back side of the ground plane can be used to heat up the VO_2_ and a laser thermometer can be used to precisely monitor the temperature. However, this results in a bulky structure. Alternatively, Platinum (Pt) resistive heater can be deposited under the VO_2_ patterns in the microfabrication process. The VO_2_ temperature can be precisely controlled by applying bias voltage to the resistive heaters^58^.

### Equivalent circuit model

**Figure 4:**
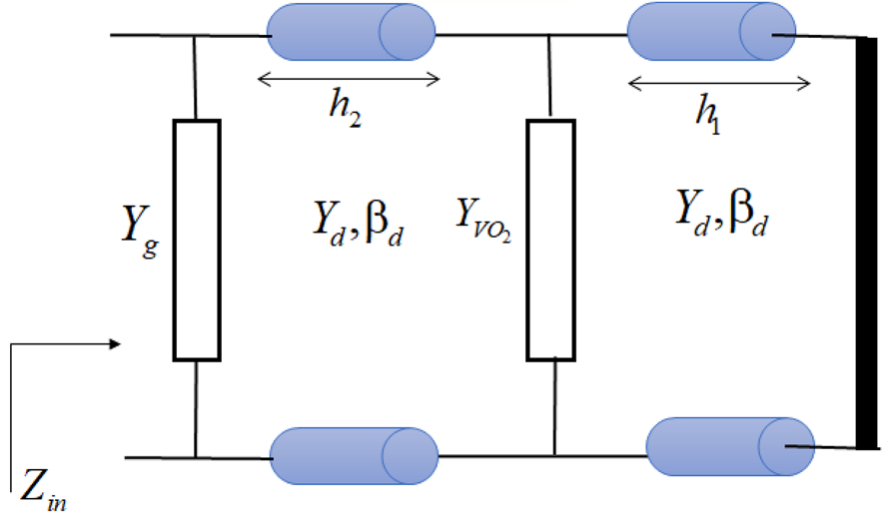
The circuit model equivalent to the proposed absorber.

An equivalent circuit model is developed and analyzed for the designed absorber in this section as plotted in Fig. 4.

In the circuit model, the VO_2_ layer including the array of cross strips is modelled as a surface admittance. For the metallic phase of the VO_2_ layer, the surface admittance can be modelled as the lumped elements in the form of a series *R*_*1*_*-L*_*1*_*-C*_*1*_. In this model, the gap between two adjacent strips is modeled as a capacitor, whereas the vertical strips for TE polarization and horizontal strips for TM polarization are modelled as inductor. The resistance is associated with the finite conductivity of the VO_2_ layer in the metallic phase. The values of the lumped elements corresponding to the surface admittance of VO_2_ cross strips are *C*_1_ = 0.19 fF, *L*_1_ = 9.13 pH, *R*_1_ = 226 Ω. Therefore, the surface admittance corresponding to VO_2_ layer can be computed as

4 $$ Y_{{VO_{2} }} = \left( {R_{1} + jL_{1} \omega + \frac{1}{{jC_{1} \omega }}} \right)^{ - 1} . $$

For insulator phase of VO_2_, the surface admittance equivalent to VO_2_ patterned layer ($Y_{{VO_{2} }}$) defined in Eq. (4) would be zero. Therefore, the *R*_*1*_*-L*_*1*_*-C*_*1*_ branch will be deleted in the circuit model for insulator phase of VO_2_. For more accuracy, the VO_2_ layer can be modelled as a transmission line equivalent a dielectric medium with relative permittivity obtained from Eq. (1) in insulator phase. However, since the thickness of VO_2_ layer is very thin, this equivalent transmission line don’t have the considerable effects on the obtained results.

The graphene layer made of square patches array is modelled as an equivalent admittance surface admittance^2^ defined as:

5 $$ Y_{g} = \left( {\sigma_{g}^{ - 1} (\omega ) + \frac{{q_{11} }}{{j\omega \varepsilon_{eff} }}} \right)^{ - 1} \left( {\frac{{S^{2} }}{{P^{2} K}}} \right), $$

in which

6 $$ K = 1.1634\,w_{g}^{2} ,\,\,S = 1.015\,w_{g}^{2} ,\,\,q = 0.2\,\pi /w_{g} ,\,\,\varepsilon_{eff} = \varepsilon_{0} (1 + \varepsilon_{r} )/2. $$

According to (3) that can be used for each non-zero Fermi energy level, Eq. (5) can be modelled as the lumped elements in the form of a series *R*_*2*_*-L*_*2*_*-C*_*2*_^2^. The lumped element values corresponding to the surface admittance of graphene patches are

7 $$ R_{2} = \frac{{P^{2} K}}{{S^{2} }}\frac{{\pi \hbar^{2} }}{{e^{2} E_{f} \tau }},\,L_{2} = \tau R_{2} ,\,C_{2} = \frac{{S^{2} }}{{P^{2} K}}\frac{{\varepsilon_{eff} }}{q}. $$

It is known that an array of metallic patches shows capacitive behavior^59^. When we use graphene for realization of the patches, it contributes an additional resistive-inductive surface admittance because of the real and imaginary parts of graphene surface conductivity. The relations of (5) and (7) show that the surface admittance equivalent to graphene patterned layer are corresponding to the parameters of graphene (the Fermi energy level and the relaxation time). In the following, we will consider two cases according to the Fermi energy level as *E*_*f*_ = 0.2 eV and *E*_*f*_ = 0 eV for graphene layer. For case of *E*_*f*_ = 0.2 eV, the lumped elements corresponding to graphene layer can be computed using (4). While for case of *E*_*f*_ = 0 eV, since we can’t use Eq. (3) for computation of the conductivity of the graphene, so the equivalent surface admittance should be computed by (5) where the conductivity is computed by (2).

As shown in the equivalent circuit model of Fig. 4, the dielectric spacers are modeled as transmission line stubs, where $\beta_{d} = k_{0} \sqrt {\varepsilon_{r} }$ and $Y_{d} = \sqrt {\varepsilon_{r} } /\eta_{0}$ are the propagation constant and the characteristic admittance of the transmission lines corresponding to the dielectric spacers respectively, where ${\eta }_{0}=120\pi$ is the free-space impedance and ${k}_{0}=\omega /c$ (*c* is the speed of light in vacuum) is the free space wave number. The metallic back reflector is considered as a short circuit. The input admittance of the proposed structure is obtained as:

8 $$ \begin{aligned}   Y_{{in}} \, &  = Y_{g}  + \left( {Y_{d} \frac{{Y_{1}  + jY_{d} \tan (\beta _{d} h_{2} )}}{{Y_{d}  + jY_{1} \tan (\beta _{d} h_{2} )}}} \right), \\    Y_{1} \, &  = Y_{{VO_{2} }}  - jY_{d} \cot (\beta _{d} h_{1} ), \\  \end{aligned}  $$

where

9 $$ \begin{gathered} Y_{g} = \left( {R_{2} + jL_{2} \omega + \frac{1}{{jC_{2} \omega }}} \right)^{ - 1} , \hfill \\ Y_{{VO_{2} }} = \left( {R_{1} + jL_{1} \omega + \frac{1}{{jC_{1} \omega }}} \right)^{ - 1} . \hfill \\ \end{gathered} $$

Finally, the absorption values can be calculated as:

10 $$ A(\omega ) = 1 - R(\omega ) = 1 - \left| {\frac{{\left( {{{Z_{in} } \mathord{\left/ {\vphantom {{Z_{in} } {Z_{0} }}} \right. \kern-0pt} {Z_{0} }}} \right) - 1}}{{\left( {{{Z_{in} } \mathord{\left/ {\vphantom {{Z_{in} } {Z_{0} }}} \right. \kern-0pt} {Z_{0} }}} \right) + 1}}} \right|^{2} $$

where $Z_{in} = Y_{in}^{ - 1}$ and $Z_{0}$ is the free-space impedance. When the real part of the normalized impedance ($Z_{in} /Z_{0}$) is approximately 1, and the imaginary part approaches near zero, the impedance matching condition occurs resulting in the maximum absorption. Comparisons between the results obtained from the circuit model and full-wave EM simulations of the absorber in HFSS are investigated in the next section.

## Results and discussion (dual narrowband-broadband absorber)

This section presents the simulation results of the switchable absorber. The multifunctinality of the proposed structure is due to the switching behavior of the VO_2_ layer from a metal phase to an insulator through the temperature transition and the electrical tunable property of the graphene surface conductivity. The geometrical parameters of the proposed structure are presented in Table 1. These parameters are designed to realize a dual-band absorber. The unit cell and the dielectric spacer dimensions are designed and optimized based on the impedance matching condition through the full-wave electromagnetic simulations in the Ansys HFSS. The process is started by designing each metamaterial layer separately and then putting them together. First, dimensions of the graphene patch unit cell and the dielectric spacer of *h*_1_ + *h*_2_ thickness is optimized to obtain a narrowband impedance matching at 0.76 THz resulting in a narrow absorption band. In the second step, dimensions of the cross-shaped VO_2_ unit cell and *h*_1_ are optimized through a separate full-wave simulation to achieve a wideband impedance matching and absorption within 2.3–5.63 THz. By optimizing *h*_1_ dimension in this step, the *h*_2_ value is automatically obtained by knowing *h*_1_ + *h*_2_ from the previous step. In the last step, the final dual-band absorber is designed by putting the two layers together and performing small dimensional optimizations to fix the absorption bands (narrow absorption at 0.76 THz and wide absorption band within 2.3–5.63 THz.

**Table 1:** The geometrical dimensions of the proposed absorber in Fig. 1 to realize a 2-bits switchable absorber.

*l*	18* µ*m	*w*_g_	34 *µ*m
*w*	2.5* µ*m	*h*_2_	2 *µ*m
*h*_vo2_	0.2 *µ*m	*h*_g_	1 nm
*h*_1_	13 *µ*m	*P*	40 *µ*m

**Figure 5:**
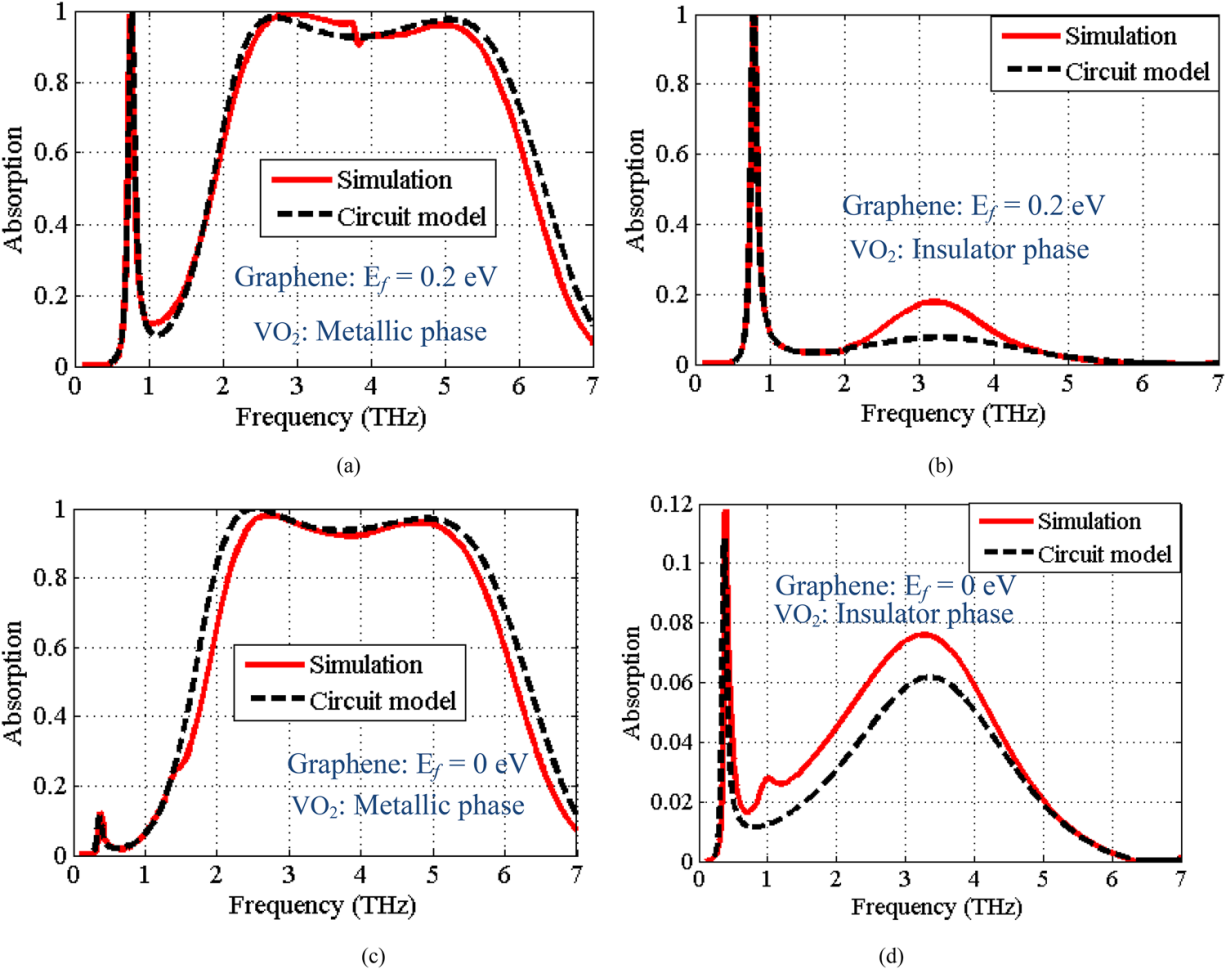
The absorption spectra of the narrowband-broadband absorber prototype, when the Fermi energy level of graphene and the phase of VO_2_ material considered as (**a**) State 11: *E*_*f*_ = 0.2 eV and metallic phase of VO_2_, (**b**) State 10: *E*_*f*_ = 0.2 eV and insulator phase of VO_2_, (**c**) State 01: *E*_*f*_ = 0 eV and metallic phase of VO_2_, and (**d**) State 00: *E*_*f*_ = 0 eV and insulator phase of VO_2_.

Figure 5a presents the absorption response of the structure with parameters presented in Table 1, when the graphene Fermi energy level is *E*_*f*_ = 0.2 eV and the VO_2_ material is in the metallic phase ($\sigma_{{VO_{2} }} = 200000\,\,S/m$). The relaxation time of graphene is considered as $\tau = 2.5\,ps$. We consider this state as 11.

As seen, the response shows two absorption bands with the fractional bandwidths (FBW) of 4 and 84% for the low frequency (0.745–0.775 THz) and high frequency (2.3–5.63 THz) bands, respectively. FBW is defined as $FBW = 2\left( {f_{h} - f_{l} } \right)/\left( {f_{h} + f_{l} } \right)$ (where $f_{h}$ and $f_{l}$ are the high and low frequencies with 90% absorption). If we consider, the absorption band above 90% as an “ON” state and the absorptivity below 20% as “OFF” state. This case represents the “ON-ON” state. In this state, the absorption spectra includes a narrow absorption band at low frequencies, where the surface plasmons of graphene play the main role to create resonant behavior (Fermi energy level of graphene is set to 0.2 eV). The broad upper absorption band is obtained at the higher frequencies due to the resonant behavior of VO_2_ layer in the metallic phase. In 10 state, we switch the VO_2_ material to an insulator, whereas the graphene Fermi energy level is unchanged. As plotted in Fig. 5b, in this case the high frequency absorption band produced by the resonance of the VO_2_ material in the metallic state is omitted and the absorber shows a single narrow absorption band at low frequencies due to the resonant behavior of graphene. In 01 state, the Fermi energy level of graphene is set to zero and the VO_2_ material is switched to the metallic phase. As expected, the low frequency absorption band is eliminated and only the high frequency absorption band remains. Finally, when the Fermi energy level is set to zero and the VO_2_ material is in insulator phase (state 00) and the absorption values are reduced to below 12% for both of the operation bands. Table 2 lists a summary of the proposed absorber functionality at four different states. Furthermore, the simulated results plotted in Fig. 5 are compared against the results obtained from the equivalent circuit model for all of the four operational states of the absorber. A very good agreement between the full-wave and the circuit model simulation results verifies the presented circuit model analysis.

**Table 2:** Summary of the absorption performance for low and high frequency bands at different operational states.

State	Graphene	VO_2_	Low frequency narrowband	High frequency wideband
11	*E*_*f*_ = 0.2 eV	Metallic phase	Absorptivity above 90% (ON)	Absorptivity above 90% (ON)
10	*E*_*f*_ = 0.2 eV	Insulator phase	Absorptivity above 90% (ON)	Absorptivity below 20% (OFF)
01	*E*_*f*_ = 0 eV	Metallic phase	Absorptivity below 15% (OFF)	Absorptivity above 90% (ON)
00	*E*_*f*_ = 0 eV	Insulator phase	Absorptivity below 12% (OFF)	Absorptivity below 8% (OFF)

**Figure 6:**
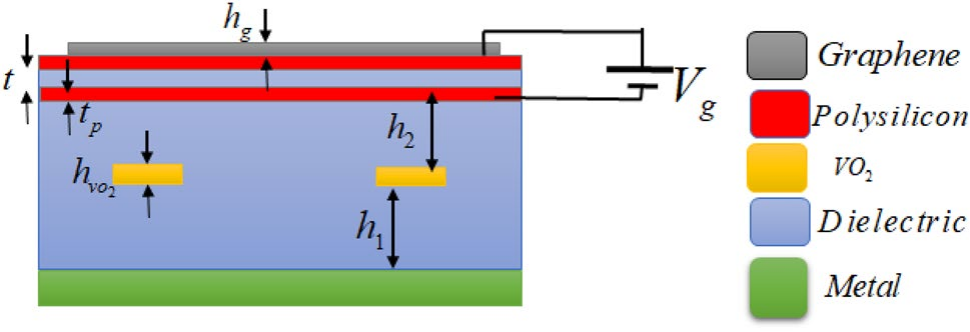
Electrostatic biasing of graphene using two polysilicon DC gating sheets. The bias voltage applied between the two ultra-thin polysilicon sheets tunes the Fermi level of the graphene patches. *t* = 50 nm is the distance between two polysilicon sheets, and *t*_p_ = 20 nm is the thicknesses of the polysilicon sheets.

It is worth to describe that the conductivity of graphene can be tuned by controlling the Fermi level through applying a DC bias voltage. For electrostatic biasing of patterned graphene layer, one may use polysilicon DC gating sheets^33,60–63^ or ion-gel layer^64,65^. We present a way of implementation utilizing polysilicon DC gating sheets. An example of DC biasing using two polysilicon DC gating sheets of distance *t* = 50 nm is shown in Fig. 6. The bias voltage is applied to the polysilicon sheets to modulate the Fermi level of the graphene patches. Since the graphene in the proposed structure includes a non-continuing pattern, it is required to use two polysilicon sheets as reported in Ref.^52^. The graphene layer should be deposited on the top polysilicon sheet. To suppress the effect of polysilicon sheets on the electromagnetic response of the device, the polysilicon sheets should be very thin (20 nm)^33^. The approximate relation between the graphene Fermi level and the extra bias voltage (*V*_g_) can be expressed as $E_{f} = \hbar v_{f} \sqrt {\frac{{\pi \varepsilon_{0} \varepsilon_{r} V_{g} }}{et}}$, where $v_{f}$ is the Fermi velocity.

**Figure 7:**
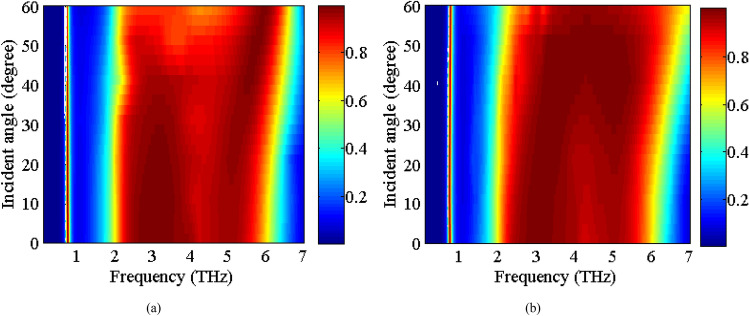
The Absorption spectra of the proposed absorber in state 11 as function of different incident angles for (**a**) TE polarization, (**b**) TM polarization.

Now, we study the stability of the absorption spectra under oblique incidences of the electromagnetic waves. The scan angle performance of an absorber is very critical in practical applications. Figure 7 indicates the absorption spectra under the oblique incident angles up to 60° for both of the TE and TM polarizations. It is expected that the absorber would have excellent stability under different polarization angles from 0° to 90° due to the geometrical symmetry of the absorber unit cell. The absorption level remains larger than 90% in the TE polarization for low absorption band up to 55° incidence angle, whereas this is the case for the high frequency absorption band up to 50°. This can be explained due to a decrease in the electric dipole resonance of the VO_2_ layer due to the reduction of the tangential component of the electric field for larger incidence angles. For TM polarization, the absorptivity remains larger than 90% up to 60° oblique incidence for both of the absorption bands.

In the following, we compare the proposed multifunctional absorber with other absorbers composed of hybrid metamaterial of graphene and VO_2_. Although the various absorbers composed of graphene or VO_2_ metamaterials are presented in the literature. Here, we just survey the absorbers that are composed of both graphene and VO_2_. Hence the switchable behavior of the absorbers is obtained by means of two control factors being (i) the switching behavior of the VO_2_ layer from a metal phase to an insulator through the temperature transition and (ii) the electrical tunable property of the graphene surface conductivity by changing the Fermi energy level. Comparisons between the absorbers in Refs.^46–48^ and the proposed absorber are presented in Table 3. Based on the table, all of the previous designs show three switchable states, whereas the proposed absorber offers four switchable operational states.

**Table 3:** Comparison of the proposed absorber to other absorbers composed of hybrid metamaterial of graphene and VO_2_.

Reference	Structure stack up	Number of states	Operation states
^46^	The VO_2_ resonant ring, a graphene ring beneath VO_2_, an insulator layer and metallic layer	3	Single broadband/single narrowband/ No absorption
^47^	The VO_2_ square loops, a square-shaped graphene layer, the polysilicon sheet, the first polyethylene cyclic olefin copolymer (Topas) layer, the VO_2_ film, the second Topas layer, and a gold (Au) film	3	Single high-frequency broadband/single low-frequency broadband/No absorption
^48^	The graphene pattern composed of four hollow, the polymide layer, the vanadium dioxide pattern, a polymide layer and a metallic film	3	Single narrowband/dual narrowband/single broadband
This work (first design)	The graphene patch, dielectric (COC) layer, four VO_2_ cross strips, dielectric (COC) layer, metallic film	4	Dual band/single lower band/single higher band/No absorption

## Dual broadband switchable absorber

In this section, we show that the proposed absorber configuration can also be optimized to achieve dual broadband absorption response. Generally, impedance matching condition can be used for designing absorbers meaning that the input impedance of the absorber should match the free-space impedance within the bandwidth of the absorber (Im(Z_in_) = 0, and Re{Z_in_} = 377 Ω). In order to achieve broadband absorption, in addition to the impedance matching condition, the derivative of the imaginary part of the impedance is also set to zero within the absorption band^66^. This is performed by optimizing the thickness of the dielectric spacer between the graphene and the VO_2_ layers and the relaxation time of graphene. In the dual broadband absorber, the thickness of the top dielectric spacer and the relaxation time of graphene are *h*_2_ = 50 µm and *τ* = 0.25 ps, whereas all of the other parameters are unchanged compared with the initial design.

**Figure 8:**
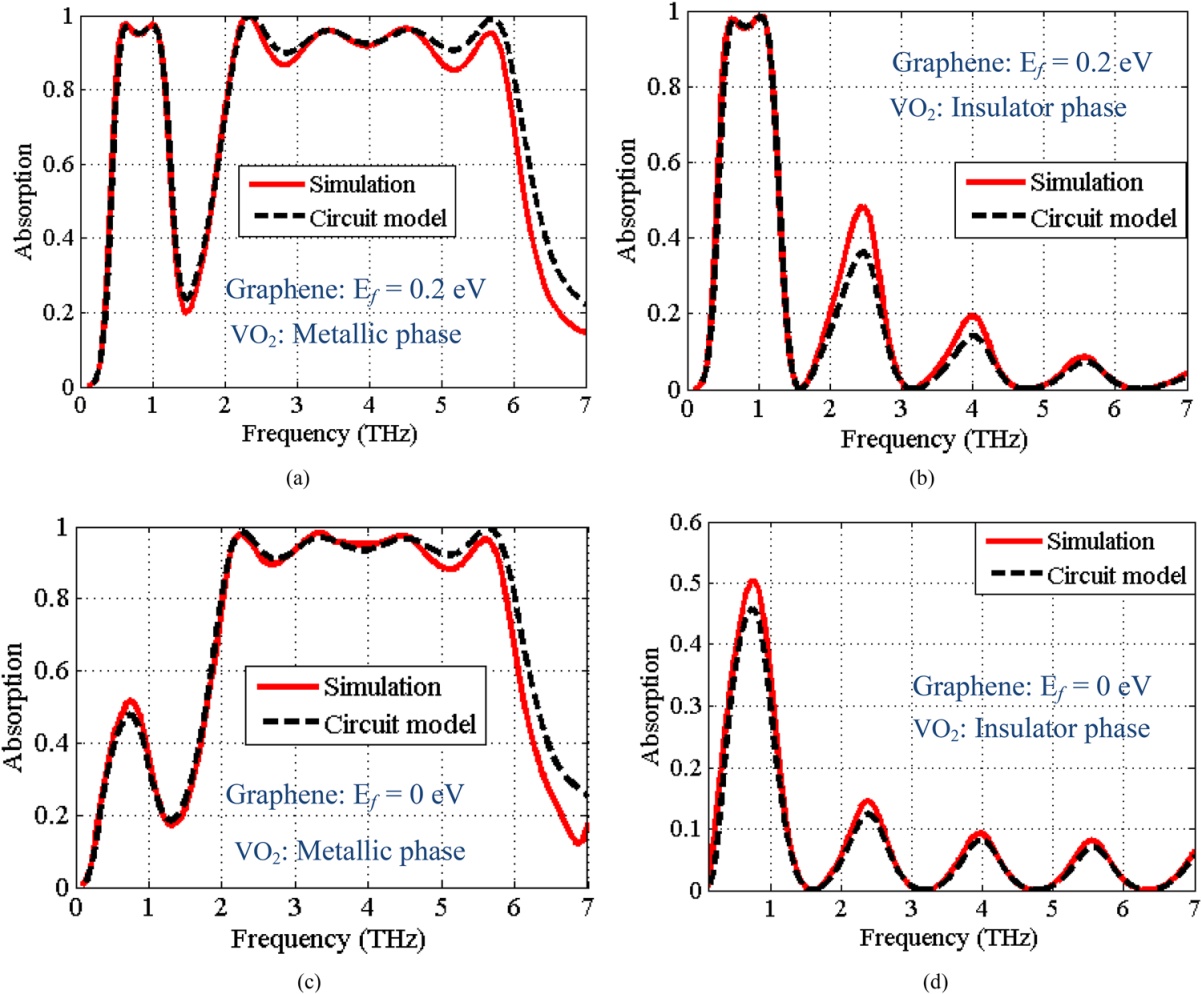
The absorption spectra of the proposed structure, where the Fermi energy level of graphene and the phase of VO_2_ material considered as (**a**) state 11: *E*_*f*_ = 0.2 eV and metallic phase of VO_2_, (**b**) State 10: *E*_*f*_ = 0.2 eV and insulator phase of VO_2_, (**c**) State 01: *E*_*f*_ = 0 eV and metallic phase of VO_2_, and (**d**) State 00: *E*_*f*_ = 0 eV and insulator phase of VO_2_.

Figure 8 shows the absorption spectra of the new design for the four states. According to Fig. 8a, in state 11, the absorption spectra includes two absorption bands, where the surface plasmons of graphene play the main role in creating resonant behavior of the low absorption band (Fermi energy level of graphene is set to 0.2 eV) and upper absorption band is due to the resonant behavior of VO_2_ layer in the metallic phase. The fractional bandwidths (FBWs) are 69% and 93.5% for low frequency (0.55–1.12 THz) and high frequency (2.15–5.93 THz) bands, respectively. Similar to the previous design, the absorption can be switched by adjusting the Fermi energy level of graphene to zero and switching the VO_2_ material to the insulating phase. The absorption responses for the other three operational states of the absorber are plotted in Fig. 8b–d confirming four state switching performance of the absorber. In addition, very good agreements between the full-wave and circuit model simulation results in all four states verify the developed circuit model. The derived values of the lumped elements for this design are similar to the previous section except the value of the resistance corresponding to the surface admittance of graphene that is different because of the change in the graphene relaxation time.

As seen, the absorption of level in State 00 (Fig. 8d) is larger in comparison to Fig. 5d. In the second design we changed two parameters (relaxation time of graphene and the thickness of the dielectric spacer between the VO_2_ and graphene layers (*h*_2_)). According to the circuit model presented in Section "Equivalent circuit model", these parameters mainly effect the surface admittance corresponding to the graphene and the characteristic admittance of the transmission line corresponding to the dielectric spacer, respectively. Thus, the input impedance of the structure is modified resulting in higher absorption values respect to the first design in Sate 00. In addition, increasing the thickness of the dielectric spacer between the VO_2_ and graphene layers in the second design excites the higher order Fabry–Pérot resonances. Indeed, the Fabry–Perot resonances formed between the graphene at the top layer and the VO_2_ at the bottom layer results in significant changes as seen in Fig. 8b, while small thickness of dielectric spacer in first design suppresses these resonances.

**Figure 9:**
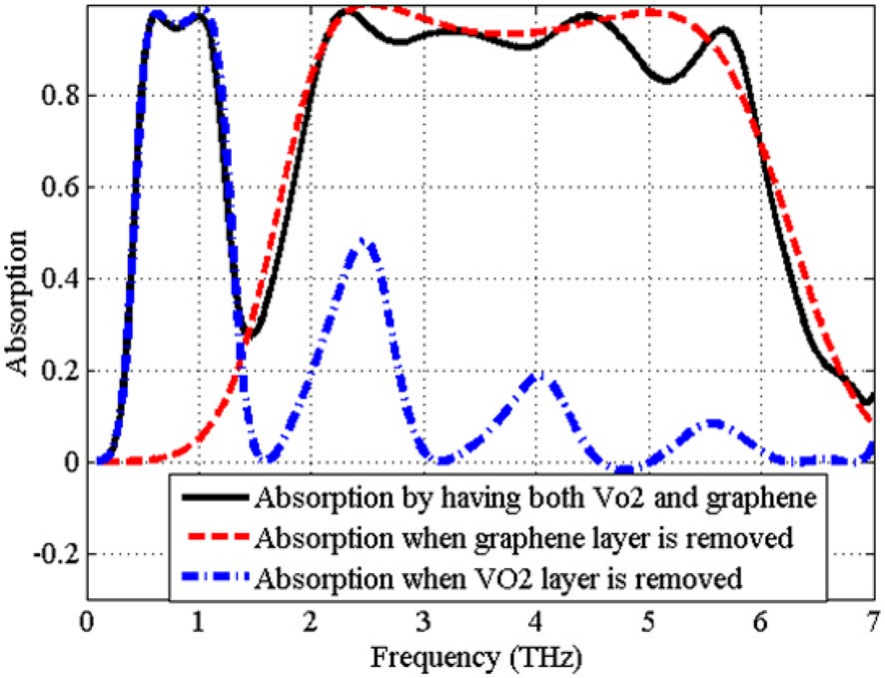
Full-wave simulated absorption, when the graphene layer is removed, when the VO_2_ layer is removed, and when both of the VO_2_ and graphene layers are included.

Based on the results in Fig. 8, there are ripples in the upper absorption band. Note that the dielectric spacer between graphene and VO_2_ (*h*_2_ thickness) is thicker in the second design. The ripples are artifacts of the graphene layer and the thick *h*_2_ dielectric spacer. In fact, the combination of the graphene layer and the dielectric spacer produces higher order Fabry–Perot resonances causing ripples in the upper absorption band. This is confirmed by removing the top graphene layer from the absorber and simulating it with just VO_2_. We also considered the case, where the VO_2_ layer is removed and just the graphene layer is included. The full wave simulated absorptions for these cases are presented in Fig. 9. It is clear from the curves in Fig. 9 that the VO_2_ absorption band is almost flat if the graphene and the upper dielectric spacer are removed from the structure. Furthermore, the plot shows the higher-order resonances for the graphene only absorber, when the VO_2_ is removed.

**Figure 10:**
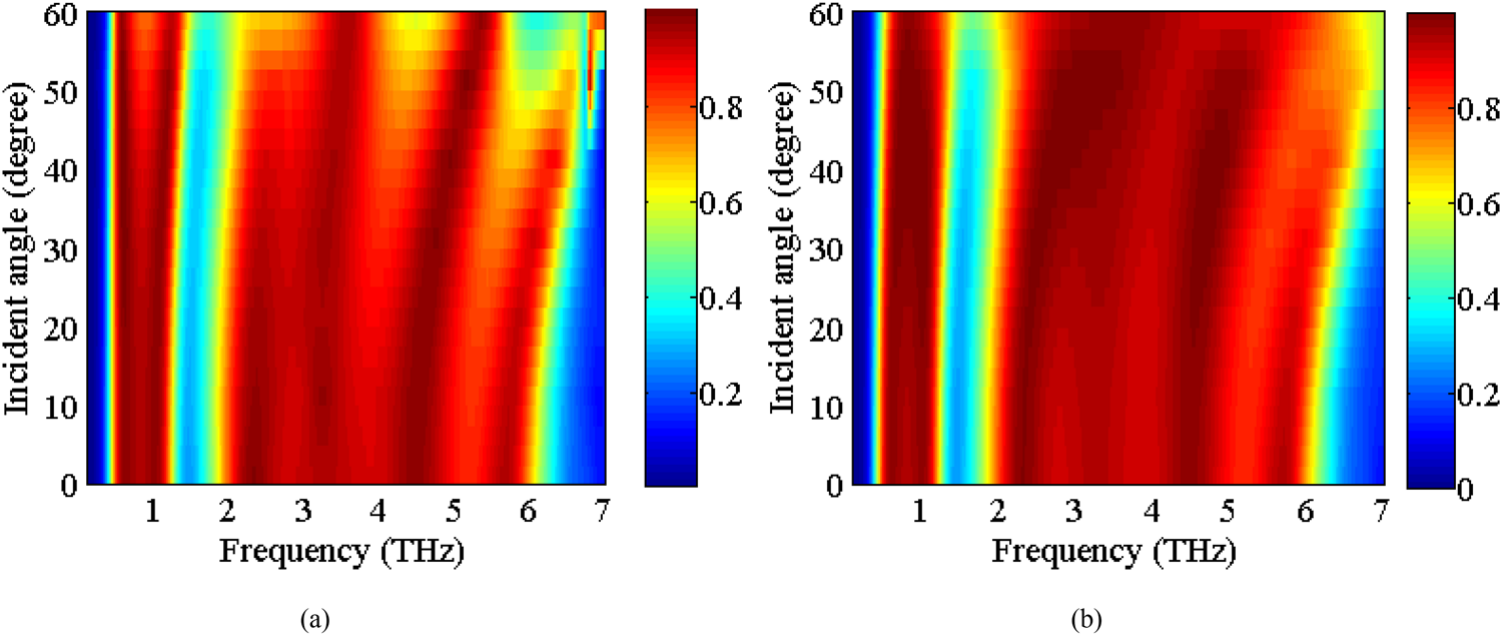
The absorption spectra of the proposed absorber in state (11) as function of different incident angles for (**a**) TE polarization, (**b**) TM polarization.

The stability of the absorption spectra under the oblique incident angles (up to 60°) is studied in Fig. 10 for the TE and TM polarizations. As observed, the absorptivity remains larger than 90% up to 50° oblique incidence for both TE and TM polarization in both the absorption bands.

**Figure 11:**
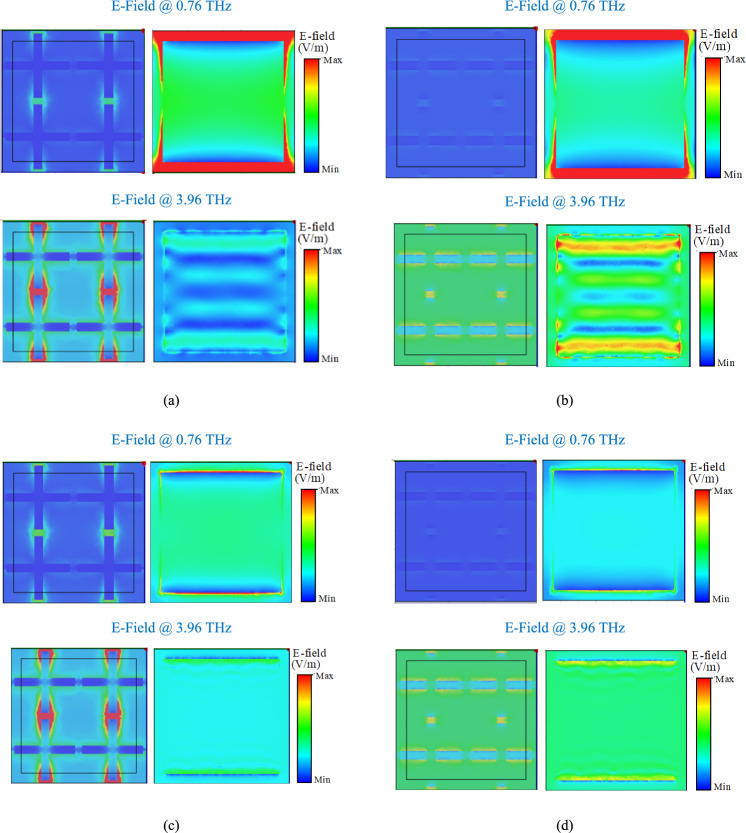
Electric field distributions on the VO_2_ cross resonators and graphene patches in four operational states. (**a**) State 11, (**b**) sate 10, (**c**) state 01, and (**d**) state 00. The field distributions are plotted at 0.76 THz and 3.96 THz that are the central frequencies of the lower and upper absorption bands.

**Figure 12:**
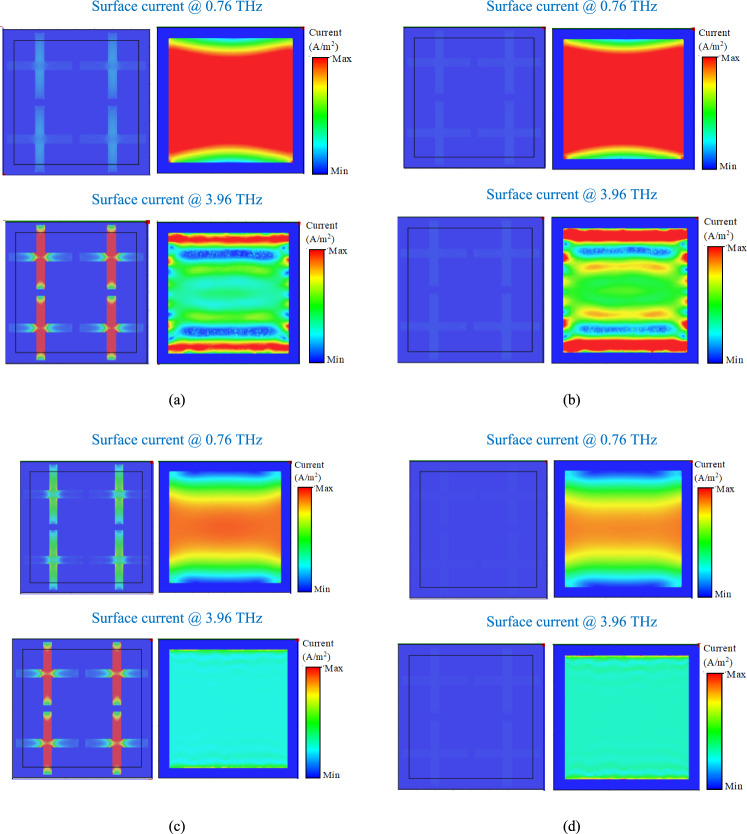
Surface current distributions on the VO_2_ cross resonators and graphene patches in four operational states. (**a**) State 11, (**b**) sate 10, (**c**) state 01, and (**d**) state 00. The field distributions are plotted at 0.76 THz and 3.96 THz that are the central frequencies of the lower and upper absorption bands.

Finally, to better understand the performance of proposed structure, the E-field and surface current distributions in four different operational states of the absorber are obtained and plotted in Figs. 11 and 12. The field distributions are plotted at 0.76 THz and 3.96 THz that are the center frequencies of the first and second absorption bands for the TE excitation mode.

Based on the Results in Fig. 11, the E-field distribution is maximum at the edges of the VO_2_ cross-shaped resonators at 3.96 THz in States 11 and 01 meaning that the VO_2_ mainly contributes to the upper absorption band. The E-field distribution is maximum at the top and bottom edges of the graphene patches at 0.76 THz in States 11 and 10 meaning that the graphene patches mainly contribute to the first absorption band. In addition, there is no significant E-field distribution on the edges of the VO_2_ crosses at 0.76 THz in any of the states meaning that VO_2_ does not contribute to the lower absorption band. Also, there is no significant E-field distribution on the graphene patches at 3.96 THz in any of the states meaning that the graphene patches do not contribute to the upper absorption band. The surface current distributions in Fig. 12 agrees with the results in Fig. 11 meaning that the surface current is maximum on the VO_2_ cross resonators at 3.96 THz in Sates 11 and 01, while there is no significant current distribution on the VO_2_ pattern at 0.76 THz in any of the operational states. Furthermore, the surface current is maximum on the graphene patches at 0.76 THz in States 11 and 10, while there is no significant current distribution on the graphene layer at 3.96 THz in any other states.

## Conclusion

Dual narrowband-wideband and dual wideband switchable absorbers based on a hybrid metamaterial formed by a combination of graphene-vanadium dioxide have been investigated in this paper. The proposed absorbers are composed of two stacked resonator arrays, including graphene patches and VO_2_ cross strips. The stacked resonator arrays were placed on a dielectric spacer terminated by a metallic film as a back reflector for the EM waves. The proposed structures offer dual absorption bands, where the lower absorption band is produced by the resonance of the graphene layer and the high frequency absorption band is realized by the resonance behavior of the VO_2_ layer in the metallic phase. Two different designs were studied in this paper. The first design produces narrow-wide absorption bands, and the second prototype offers dual wide absorption bands. It was shown that the absorption spectra of the structures can be switched by controlling the Fermi energy level of graphene and the operational phase of VO_2_ layers. Such a characteristic creates four different operational states for the designed absorbers: dual-band absorption, single-band low frequency absorption, single-band high frequency absorption, and no absorption states. Such a device would be a potential candidate for application in the switchable and reconfigurable terahertz measurement and communication systems. The designed absorbers are polarization insensitive and provide high stability over a wide range of incident angles and offer advantages such as simple structure, high absorption, both narrow and wide bandwidth characteristics. The proposed four state switchable absorber may have promising application in spectrum multiplexing for the reconfigurable communication, sensing or imaging systems within the terahertz regime, where a special frequency band can be selectively absorbed (filtered) using the proposed absorber and the spectrum selection can be switched by controlling the VO_2_ and graphene patterns.

## Data Availability

The data that support the findings of this study are available from the corresponding author on reasonable request.

## References

[1] Biabanifard M, Arsanjani A, Abrishamian MS, Abbott D (2020). Tunable terahertz graphene-based absorber design method based on a circuit model approach. IEEE Access.

[2] Barzegar-Parizi S, Ebrahimi A, Ghorbani K (2021). Dual-broadband and single ultrawideband absorbers from the terahertz to infrared regime. J. Opt. Soc. Am. B.

[3] Ye L, Chen X, Zeng F, Zhuo J, Shen F, Liu QH (2019). Ultrawideband terahertz absorption using dielectric circular truncated cones. IEEE Photon. J..

[4] Nourbakhsh M, Zareian-Jahromi E, Basiri R, Mashayekhi V (2020). An ultra-wideband terahertz metamaterial absorber utilizing sinusoidal-patterned dielectric loaded grapheme. Plasmonics.

[5] Barzegar-Parizi S, Ebrahimi A, Ghorbani K (2020). High-Q dual-band graphene absorbers by selective excitation of gra- pheneplasmonpolaritons: Circuit model analysis. Opt. Laser Technol..

[6] Biabanifard M, Asgari S, Biabanifard S, Abrishamian MS (2019). Analytical design of tunable multi-band terahertz absorber composed of graphene disks. Optik.

[7] Barzegar-Parizi S, Ebrahimi A (2021). Terahertz high-Q absorber based on holes array perforated into a metallic slab. Electronics.

[8] Xiong H, Ma X, Liu H, Xiao D, Zhang H (2023). Research on electromagnetic energy absorption and conversion device with four-ring multi-resistance structure. Appl. Phys. Lett..

[9] Deng J-H, Xiong H, Yang Q, Suo M, Xie J-Y, Zhang H-Q (2023). Metasurface-based microwave power detector for polarization angle detection. IEEE Sensors J..

[10] Atwater HA, Polman A (2011). Plasmonics for improved photovoltaic devices. Nat. Mater..

[11] Bagmanci M, Karaaslan M, Unal E, Akgol O, Bakır M, Sabah C (2019). Solar energy harvesting with ultra-broadband metamaterial absorber. Int. J. Mod. Phys..

[12] Liu X, Tyler T, Starr T, Starr AF, Jokerst NM, Padilla WJ (2011). Taming the blackbody with infrared metamaterials as selective thermal emitters. Phys. Rev. Lett..

[13] Morden D, Smith EM, Avrutsky I, Hendrickson JR, Agha I, Vangal Sh (2022). Tunable angle-independent mid-infrared optical filters using GST-based micro resonator arrays. Opt. Mater. Express.

[14] Barzegar-Parizi S, Ebrahimi A (2023). Terahertz all metallic perfect absorber for refractive index sensing and glucose concentration detection. Phys. Scripta.

[15] Barzegar-Parizi S (2023). Refractive index sensor with dual sensing bands based on array of Jerusalem cross cavities to detect the hemoglobin concentrations. Opt. Quantum Electron..

[16] Xiao S, Zhu X, Li B, Asger N (2016). Graphene-plasmon polaritons: From fundamental properties to potential applications. Front. Phys..

[17] Bao Q, Loh KP (2012). Graphene photonics, plasmonics, and broadband optoelectronic devices. ACS Nano.

[18] Wang F, Wang F, Zhang Y, Tian Ch, Girit C, Zettl A, Crommie M, Shen YR (2008). Gate-variable optical transitions in graphene. Science.

[19] Koppens FH, Chang DE, de Abajo FJG (2011). Graphene plasmonics: A platform for strong light-matter interactions. Nano Lett..

[20] Barzegar-Parizi S, Vafapour Z (2023). Dynamically switchable Sub-THz absorber using VO2 metamaterial suitable in optoelectronic applications. IEEE Trans. Plasma Sci..

[21] Jiang H, Wang Y, Cui Z, Zhang X, Zhu Y, Zhang K (2022). Vanadium dioxide-based terahertz metamaterial devices switchable between transmission and absorption. Micromachines.

[22] He J, Zhang M, Shu Sh, Yan Y, Wang M (2020). VO_2_ based dynamic tunable absorber and its application in switchable control and real-time color display in the visible region. Opt. Express.

[23] Pu M, Wang M, Hu Ch, Huang Ch, Zhao Z, Wang Y, Luo X (2012). Engineering heavily doped silicon for broadband absorber in the terahertz regime. Opt. Express.

[24] Wu B, Wang M, Wu X (2021). Broadband tunable absorption based on phase change materials. Results Phys..

[25] Linyang G, Xiaohui M, Zhaoqing Ch, Chunlin X, Jun L, Ran Zh (2021). Tunable a temperature-dependent GST-based metamaterial absorber for switching and sensing applications. J. Mater. Res. Technol..

[26] Guo Z, Yang X, Shen F (2018). Active-tuning and polarization-independent absorber and sensor in the infrared region based on the phase change material of Ge_2_Sb_2_Te_5_ (GST). Sci. Rep..

[27] Buchnev O, Podoliak N, Kaczmarek M, Zheludev NI, Fedotov VA (2015). Electrically controlled nanostructured metasurface loaded with liquid crystal: Toward multifunctional photonic switch. Adv. Opt. Mater.

[28] Lee C, Huang C, Liu H, Zhang X, Liu Z (2013). Resonance enhancement of terahertz metamaterials by liquid crystals/indium tinoxide interfaces. Opt. Express.

[29] Xia F, Mueller T, Lin Y, Valdes-Garcia A, Avouris P (2009). Ultrafast graphene photodetector. Nat. Nanotechnol..

[30] Ding Y, Zhu X, Xiao S, Hu H, Frandsen LH, Mortensen NA, Yvind K (2015). Effective electro-optical modulation with high extinction ratio by a graphene-silicon microring resonator. Nano Lett..

[31] Terrones H, Lv R, Terrones M, Dresselhaus MS (2012). The role of defects and doping in 2D graphene sheets and 1D nanoribbon. Rep. Prog. Phys..

[32] Mak KF, Sfeir MY, Wu Y, Lui CH, Misewich JA, Heinz TF (2008). Measurement of the optical conductivity of graphene. Phys. Rev. Lett..

[33] Ye L, Chen X, Cai G, Zhu J, Liu N, Liu QH (2018). Electrically tunable broadband terahertz absorption with hybrid-patterned graphene metasurfaces. Nanomaterials.

[34] Ye L, Chen Y, Cai G, Liu N, Zhu J, Song Z, Liu QH (2017). Broadband absorber with periodically sinusoidally-patterned graphene layer in terahertz range. Opt. Express.

[35] Barzegar-parizi S, Ebrahimi A, Ghorbani K (2023). Terahertz wideband modulator devices using phase change material switchable frequency selective surfaces. Phys. Scr..

[36] Zhao Y, Huang Q, Cai H, Lin X, Lu Y (2018). A broadband and switchable VO2 -based perfect absorber at the THz frequency. Opt. Commun..

[37] Zou M, Yi L, Zhao W, Zhang X, Wu Y, Peng Ch, Fan L, Li J, Yan J, Zhuang J, Mei J, Wang X (2021). Dynamically tunable perfect absorber based on VO_2_–Au hybrid nanodisc array. Opt. Eng..

[38] Luo H, Liu H, Chen C, Feng Y, Gao P, Ren ZY, Qiao YJ (2022). Dual-broadband terahertz absorber based on phase transition characteristics of VO2. Results Phys..

[39] Liu H, Wang ZH, Li L, Fan Y-X, Tao ZY (2019). Vanadium dioxide assisted broadband tunable terahertz metamaterial absorber. Sci. Rep..

[40] Wu GZ, Jiao XF, Wang YD, Zhao ZP, Wang YB, Liu JG (2021). Ultra-wideband tunable metamaterial perfect absorber based on vanadium dioxide. Opt. Express.

[41] Mou N, Tang B, Li J, Dong H, Zhang L (2022). Switchable ultra-broadband terahertz wave absorption with VO2-based metasurface. Sci. Rep..

[42] Huang J, Li J, Yang Y, Li J, Li J, Zhang Y, Yao J (2020). Broadband terahertz absorber with a flexible, reconfigurable performance based on hybrid-patterned vanadium dioxide metasurfaces. Opt. Express.

[43] Li Y, Gao W, Guo L, Chen Z, Li Ch, Zhang H, Jiao J, An B (2021). Tunable ultra-broadband terahertz perfect absorber based on vanadium oxide metamaterial. Opt. Express.

[44] Vassalini I, Alessandri I, de Ceglia D (2021). Stimuli-responsive phase change materials: optical and optoelectronic applications. Materials.

[45] Ruiz de Galarreta C, Carrillo SGC, Au YY, Gemo E, Trimby L, Shields J, Humphreys E, Faneca J, Cai L, Baldycheva A, Bertolotti J, Wright CD (2020). Tunable optical metasurfaces enabled by chalcogenide phase-change materials: from the visible to the THz. J. Opt..

[46] Zhang B, Xu K (2021). Dynamically switchable terahertz absorber based on a hybrid metamaterial with vanadium dioxide and graphene. J. Opt. Soc. Am. B.

[47] Liu Y, Huang R, Ouyang Zh (2021). Terahertz absorber with dynamically switchable dual-broadband based on a hybrid metamaterial with vanadium dioxide and graphene. Opt. Express.

[48] Zhang J, Yang X, Huang X, Li F, Liu P, Fu K (2021). Dynamically controllable terahertz absorber based on a graphene-vanadium dioxide-metal configuration. Superlattices Microstruct..

[49] Wang T, Zhang Y, Zhang H, Cao M (2020). Dual-controlled switchable broadband terahertz absorber based on a graphene-vanadium dioxide metamaterial. Opt. Mater. Express.

[50] Yang Q, Xiong H, Deng J-H, Wang B-X, Peng W-X, Zhang H-Q (2023). Polarization-insensitive composite gradient-index metasurface array for microwave power reception. Appl. Phys. Lett..

[51] Li D, He Sh, Su L, Du H, Tian Y, Gao Z, Xie B, Huang G (2024). Switchable and tunable terahertz metamaterial absorber based on graphene and vanadium dioxide. Opt. Mater..

[52] Barzegar-Parizi S, Khavasi A (2019). Designing dual-band absorbers by graphene/metallic metasurfaces. IEEE J. Quantum Electron..

[53] Zhang C, Zhou G, Wu J, Tang Y, Wen Q, Li S, Han J, Jin B, Chen J, Wu P (2019). Active control of Terahertz waves using vanadium-dioxide-embedded metamaterials. Phys. Rev. Appl..

[54] Xiong Y, Wen QY, Chen Z, Tian W, Wen TL, Jing YL, Yang QH, Zhang HW (2014). Simple method preparation for ultrathin VO2 thin film and control: Nanoparticle morphology and optical transmittance. J. Phys. D.

[55] Ameen S, Shaheer Akhtar M, Shin H-S (2020). Graphene Production and Application.

[56] Xu R, Bing P, Yan X, Yao H, Liang L, Li Z, Wang Z, Hu X, Wang M, Yao J (2023). Graphene-assisted electromagnetically induced transparency-like Terahertz Metabiosensor for ultra-sensitive detection of ovalbumin. Photonics.

[57] Xu W, Xie L, Zhu J, Tang L, Singh R, Wang Ch, Ma Y, Chen H-T, Ying Y (2019). Terahertz biosensing with a graphene-metamaterial heterostructure platform. Carbon.

[58] Anagnostou DE, Torres D, Teeslink TS, Sepulveda N (2020). Vanadium Dioxide for reconfigurable antennas and microwave devices: Enabling RF reconfigurability through smart materials. IEEE Antennas Propag. Mag..

[59] Luukkonen O, Simovski C, Granet G (2008). Simple and accurate analytical model of planar grids and high-impedance surfaces comprising metal strips or patches. IEEE Trans. Antennas Propag..

[60] Huang X, Hu Z, Liu P (2014). Graphene based tunable fractal Hilbert curve array broadband radar absorbing screen for radar cross section reduction. AIP Adv..

[61] Ye L, Chen Y, Cai G, Liu N, Zhu J, Song Z, Liu QH (2017). Broadband absorber with periodically sinusoidally-patterned graphene layer in terahertz range. Opt. Express.

[62] Gomez-Diaz J, Moldovan C, Capdevila S (2015). Self-biased reconfigurable graphene stacks for terahertz plasmonics. Nat. Commun..

[63] Goyal R, Vishwakarma DK (2018). Design of a graphene-based patch antenna on glass substrate for high-speed terahertz communications. Microw. Opt. Techn. Lett..

[64] Khattak MI, Ullah Z, Al-Hasan M, Sheikh F (2020). Enhanced tunable plasmonic resonance in crumpled graphene resonators loaded with gate tunable metamaterials. Opt. Express.

[65] Yunping Q, Zhou Z, Shi Q, Wen Y, Wang L, Zhao Sh, Zhang Sh, Wang X (2024). Dual-function tunable metasurface for polarization-insensitive electromagnetic induction transparency and dual-band absorption. Nanotechnology.

[66] Barzegar-Parizi S (2018). Realization of wide-angle and wideband absorber using metallic and graphene-based metasurface for mid-infrared and low THz frequency. Opt. Quantum Electron..

